# Individual and Neighborhood Stressors, Air Pollution and Cardiovascular Disease

**DOI:** 10.3390/ijerph15030472

**Published:** 2018-03-08

**Authors:** Marnie F. Hazlehurst, Paula S. Nurius, Anjum Hajat

**Affiliations:** 1 Department of Environmental & Occupational Health Sciences and Department of Epidemiology, School of Public Health, University of Washington, Seattle, WA 98195, USA; 2 School of Social Work, University of Washington, Seattle, WA 98195, USA; nurius@uw.edu; 3 Department of Epidemiology, School of Public Health, University of Washington, Seattle, WA 98195, USA; anjumh@uw.edu

**Keywords:** adverse childhood experiences, air pollution, neighborhood deprivation, cardiovascular disease

## Abstract

Psychosocial and environmental stress exposures across the life course have been shown to be relevant in the development of cardiovascular disease (CVD). Assessing more than one stressor from different domains (e.g., individual and neighborhood) and across the life course moves us towards a more integrated picture of how stress affects health and well-being. Furthermore, these individual and neighborhood psychosocial stressors act on biologic pathways, including immune function and inflammatory response, which are also impacted by ubiquitous environmental exposures such as air pollution. The objective of this study is to evaluate the interaction between psychosocial stressors, at both the individual and neighborhood level, and air pollution on CVD. This study used data from the 2009–2011 Behavioral Risk Factor Surveillance System (BRFSS) from Washington State. Adverse childhood experiences (ACEs) measured at the individual level, and neighborhood deprivation index (NDI) measured at the zip code level, were the psychosocial stressors of interest. Exposures to three air pollutants—particulate matter (both PM_2.5_ and PM_10_) and nitrogen dioxide (NO_2_)—were also calculated at the zip code level. Outcome measures included several self-reported CVD-related health conditions. Both multiplicative and additive interaction quantified using the relative excess risk due to interaction (RERI), were evaluated. This study included 32,151 participants in 502 unique zip codes. Multiplicative and positive additive interactions were observed between ACEs and PM_10_ for diabetes, in models adjusted for NDI. The prevalence of diabetes was 1.58 (95% CI: 1.40, 1.79) times higher among those with both high ACEs and high PM_10_ compared to those with low ACEs and low PM_10_ (*p*-value = 0.04 for interaction on the multiplicative scale). Interaction was also observed between neighborhood-level stressors (NDI) and air pollution (NO_2_) for the stroke and diabetes outcomes on both multiplicative and additive scales. Modest interaction was observed between NDI and air pollution, supporting prior literature on the importance of neighborhood-level stressors in cardiovascular health and reinforcing the importance of NDI on air pollution health effects. ACEs may exert health effects through selection into disadvantaged neighborhoods and more work is needed to understand the accumulation of risk in multiple domains across the life course.

## 1. Introduction

Research to date has shown the importance of childhood as a critical period in shaping development and health across the lifespan. Chronic (i.e., toxic) stress in childhood is linked to increased risk of socioeconomic disadvantage, deleterious health behaviors, and poor health outcomes in adulthood, while poor health in adulthood may be exacerbated by the presence of concurrent stressors [1]. This cumulative disadvantage across the life course may be particularly relevant for long-term disease processes such as the development of cardiovascular disease (CVD) [2]. Exposures in the physical environment, such as air pollution, also contribute to the development of CVD [3,4,5,6] and socioeconomically disadvantaged populations may be more vulnerable to these cardiovascular health impacts of air pollution [7,8]. Both psychosocial stressors and environmental exposures are socially distributed, with higher levels of both exposures among the disadvantaged, resulting in further cause for concern among these populations [9,10].

The biological mechanisms by which psychosocial stressors at the individual and neighborhood levels cause CVD may be similar to the mechanisms by which environmental hazards such as air pollution are hypothesized to cause CVD. Specifically, inflammation, epigenetic changes and impacts on the sympathetic nervous system may be pathways by which both psychosocial stressors and air pollution result in poor cardiovascular health [11,12,13,14,15,16]. Adversity early in life influences epigenetic regulation of both inflammatory and immune processes, setting the stage for subsequent health trajectories [17]. Exposures to physical toxins such as air pollution may disrupt these same biological processes, potentially through epigenetic modification, autonomic nervous system imbalance or through systemic inflammation resulting from spillover of localized inflammation in the lungs [4,15]. Differential health risk and vulnerability from factors such as psychosocial stress and socioeconomic position combined with environmental hazards is argued to be a critical but under assessed public health issue [18].

The assessment of more than one psychosocial or physical environmental stressor from both individual and neighborhood level contexts and from different points over the life course moves us towards a more integrated picture of how stress affects health and well-being. This study aimed to quantify the unique and cumulative effects of air pollution, and neighborhood and individual level stressors on CVD outcomes in a multi-level context. A severe early life psychosocial stressor, adverse childhood experiences (ACEs), and a concurrent place-based stressor neighborhood deprivation, are two sources of stress that have been previously linked to CVD [19,20,21,22,23]. We explored the impact of ACEs at the individual level and disadvantage at the neighborhood level, as they interact with a ubiquitous environmental hazard, air pollution, to affect CVD. We hypothesized that the impacts of environmental exposures (air pollution) would be greater among those with higher levels of stressors (ACEs and NDI) in contrast to those with low levels of ACEs or NDI. 

## 2. Methods

### 2.1. Study Population

The Behavioral Risk Factors Surveillance System (BRFSS) is a cross-sectional random-digit-dialed telephone survey administered annually in the US [24,25]. This representative population-based survey includes non-institutionalized persons at least 18 years old who speak English or Spanish and live in a household with a working telephone. The survey is conducted by health departments in all 50 states in collaboration with the Centers for Disease Control and Prevention. Telephone-based sampling is used, where landlines with an area code within the state are selected randomly. Once an eligible household is identified, only one adult in the household is eligible to participate. Beginning in 2008, a smaller cellphone-only sample was used in addition to the landline sample. Response rates, defined as the number of complete and partial interviews divided by an estimate of the number of eligible units in the sample, ranged from 44% to 48% in 2009–2011. Data used in this study were publicly available and research did not involve interaction with participants, thus this research is exempt from IRB approval.

Data from the 2009 through 2011 Washington State BRFSS were pooled for this study. Survey weights were not applied because pooling responses across multiple years of the survey requires complex reweighting and because we were not specifically interested in population-wide inference.

BRFSS respondents report residential locations at the zip code level. Thus, both neighborhood level exposures, neighborhood deprivation index, and air pollution, were defined at the zip code.

### 2.2. Adverse Childhood Experiences (ACEs)

ACEs were measured from 11 questions dichotomized as yes or no pertaining to the following eight categories of childhood (prior to age 18) adversity: victim of verbal or emotional abuse, victim of physical abuse, victim of sexual abuse, incarcerated family member, substance abuse, mental illness, parental divorce, and witnessing domestic violence. A final ACEs score was calculated by assigning each category a value of 1 if any of the items within that category were reported or a 0 if none of the relevant items were reported, and summing across the eight categories, resulting in a final score ranging from 0 to 8. In this analysis, we compared those with an ACEs score of 0 or 1 to those with an ACEs score of 2 through 8. This dichotomization was used because the mean ACEs score was 1.55, allowing us to compare those with a score above and below the sample mean. In sensitivity analyses, we used varying cutpoints for the ACEs score, comparing those with 0 ACEs to those with 1 or more, and comparing those with 0 to 2 ACEs to those with 3 or more (see Supplementary Materials).

### 2.3. Neighborhood Deprivation Index (NDI)

The neighborhood-level social environment was assessed using data from the 2007–2011 American Community Survey [26]. Data at the zip code scale were used to calculate the NDI, with a higher value of the index representing more deprivation. Prior work by Christine et al. used principle components analysis to identify variables to be included in the creation of the NDI. That analysis was conducted using data from all census tracts in the US, starting with 21 census variables, and identified seven variables in the first factor [9,27,28]. The correlation between each of these seven variables at the zip code level was similar to the correlation between these same variables at the census tract level, thus justifying our use of this measure. Furthermore, Singh (2003) showed similarities in factor loadings when creating indices at both the census tract and zip code levels [29]. Variables in the NDI include: median household income, median housing value, and percentage of the population within the zip code with an annual household income greater than $50,000, with at least a bachelor’s degree, with at least a high school education, with a managerial or professional occupation, and with interest, dividend, or rental income. Each variable was converted to a z-score and summed. For the purposes of this study, we compared those living in high deprivation (high NDI) zip codes (>75th percentile of NDI) to those in lower deprivation (low NDI) zip codes (≤75th percentile of NDI). We chose to use the 75th percentile as the cutpoint to provide sufficient sample size in the high NDI group, while also ensuring a clear majority of participants were classified in the low NDI group in order to create a reasonable definition of “high” exposure [30,31].

### 2.4. Air Pollution

Annual average air pollution exposures were calculated using national land use and kriging models of fine particulate matter (PM_2.5_), particulate matter less than 10 µm in diameter (PM_10_), and nitrogen dioxide (NO_2_) for the years 2008 through 2011 [32,33]. When using a point-based exposure model to estimate area-level exposures, one approach used in the literature is to select the centroid of that area and predict exposure at that point [34]. However, the zip code centroid may not be representative of locations within the zip code where the majority of the population lives, especially in large zip codes where residential locations may be clustered in one portion of the zip code. In order to select a more representative location, an alternative point was randomly selected from the centroids of populated census blocks within the zip code and the entire zip code was assigned the pollution predicted at that location. Large zip codes were assigned the average of several randomly selected populated block centroids; for zip codes less than 10.8 km^2^ pollutant values at a single centroid were used, for zip codes 10.8–29.8 km^2^ pollutant values at two centroids were averaged, for zip codes 29.8–90.8 km^2^ pollutant values at three centroids were averaged, and for zip codes greater than 90.8 km^2^ pollutant values at four centroids were averaged. Cutpoints were selected based on an analysis of variability in predicted concentrations when averaging multiple block centroids within zip codes of varying sizes. 

These annual zip code air pollution exposures were then weighted to account for the fact that participants were surveyed throughout the calendar year and provide an estimate of exposure for the 365 days prior to each individual’s BRFSS survey date. For example, if a participant was interviewed on 1 June 2010, their air pollution exposure was calculated as:

2010 annual average air pollution * (151/365 days) + 2009 annual average air pollution * (214/365 days)(1)

Using the same approach as was used for dichotomizing NDI, participants with air pollutant exposures greater than the 75th percentile in this sample were considered to have high exposures; those with air pollutant exposures less than or equal to the 75th percentile were considered low exposures [31].

### 2.5. Cardiovascular Disease (CVD) Measures and Risk Factors

We examined the presence of CVD and CVD risk factors, including diabetes, stroke, myocardial infarction (MI), coronary heart disease (CHD), hypertension, and obesity [35]. Health outcomes were self-reported. In four separate questions, participants were asked if a doctor, nurse, or other health professional ever told her/him that she/he had a heart attack, angina or coronary heart disease, a stroke, or high blood pressure. Participants were not asked about high blood pressure in the 2010 survey. Borderline high or pre-hypertensive participants were not considered to have hypertension. Similarly, women who only reported high blood pressure during pregnancy and women who reported diabetes only during pregnancy were not considered hypertensive or diabetic, respectively. Obesity was defined as a body mass index (BMI) greater than or equal to 30 kg/m^2^, calculated from self-reported height and weight. 

### 2.6. Covariates

Individual-level covariates were also obtained from BRFSS, including race/ethnicity, sex, education, income, employment, and health behaviors such as smoking and alcohol use. Population density data from ACS was used to designate zip codes as urban or rural. A simple categorization approach was used for convenience; zip codes were considered urban if the population density within that zip code was greater than the median for zip codes in Washington State [36].

### 2.7. Statistical Analysis

Four of the six outcomes were considered rare (≤10%), thus using logistic regression to estimate odds ratios (ORs) approximates the risk ratio. Hypertension and obesity were common outcomes in this population (39% and 28% respectively), so we estimated prevalence ratios (PRs) for these outcomes using log binomial models. Log binomial models were used to examine interactions between NDI and air pollution on hypertension and obesity, but they did not converge when evaluating the interaction between ACEs and air pollution and thus Poisson models were used. We also explored using multi-level models but found minimal clustering of each outcome by zip code (interclass correlation coefficient ranged from 0.2% to 1.5%) and thus we felt a multi-level model was not warranted.

A staged modeling approach was used. The minimally adjusted model included age, race/ethnicity, sex, and urban versus rural zip code. A binary indicator of urbanicity was included in the model to account for strong differences in spatial distributions of air pollution and of differential cardiovascular risk in urban and rural areas. The fully adjusted model included covariates in the minimal model plus education (less than high school, high school, or some college or more), annual income (<$35,000, $35,000–$49,999 or ≥$50,000), and employment outside of the home (binary). Models of the effects of ACEs and air pollution were additionally adjusted for NDI given that NDI is likely a strong confounder of the air pollution—CVD association [8,9]. An exploratory model further adjusted for variables on the pathway between the exposures of interest and CVD outcomes, including health behaviors—smoking (current, former, or never smoker), binge drinking (binary), and exercise (binary)—and BMI except when obesity was the outcome. Results are shown only for the fully adjusted model; data from minimally adjusted models or exploratory models are available upon request. Results from main effects models for effects of ACEs and NDI are included in the Supplementary Materials.

We followed the approach of VanderWeele & Knol (2014) to present interactions [37]. Interaction analyses are presented with a single common reference group (subjects with both low air pollution exposure and low ACEs or NDI); this provides more information than solely presenting estimates within strata using multiple reference categories. Estimates of the effect of air pollution within ACEs or NDI strata can be calculated from the information provided (see Supplementary Materials).

**Figure 1:**
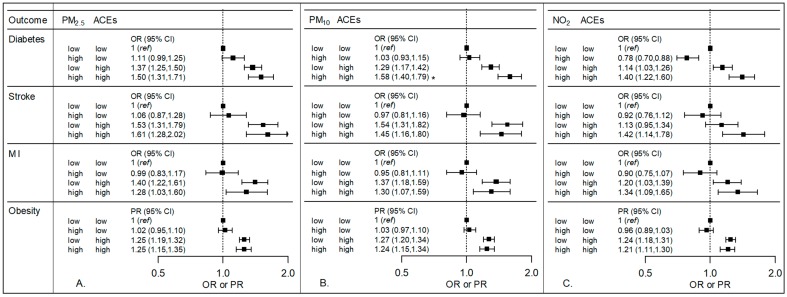
Odds ratios (OR) or prevalence ratios (PR) and 95% confidence intervals (CI) showing the relationship between air pollution, adverse childhood experiences (ACEs) and cardiovascular disease-related outcomes. The three panels show results for three pollutants: (**A**) PM_2.5_, (**B**) PM_10_, and (**C**) NO_2_. Models were adjusted for age, race, sex, urban versus rural zip code, education, income, and employment. For each pollutant and outcome combination, the reference group consists of those participants who had both low air pollution—defined as less than or equal to the 75th percentile—and low ACEs—defined as 0 or 1 ACEs. ORs or PRs for each of the other three exposure groups (high air pollution and low ACEs, low air pollution and high ACEs, and high air pollution and high ACEs) are all presented in relation to the same low-low reference group. For example, the beta coefficients for the analysis of PM_2.5_ and ACEs on diabetes were: −4.64 + (0.11)a + (0.31)b + (0.40)c, where a indicates the high-low group, b indicates the low-high, and c indicates the high-high group, corresponding to odds ratios of 1.11, 1.37, and 1.50, for the high-low, low-high, and high-high exposure groups, respectively. A *p*-value < 0.05 (noted with an asterisk) for the interaction term between with PM_10_ and ACEs was observed for diabetes.

**Figure 2:**
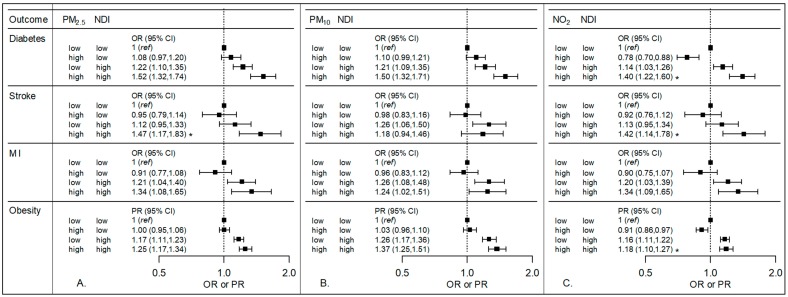
Odds ratios (OR) or prevalence ratios (PR) and 95% confidence intervals (CI) showing the relationship between air pollution, Neighborhood Deprivation Index (NDI) and cardiovascular disease-related outcomes. The three panels show results for three air pollutants: (**A**) PM_2.5_, (**B**) PM_10_, and (**C**) NO_2_. Models were adjusted for age, race, sex, urban versus rural zip code, education, income, and employment. For each outcome, the reference group consists of those participants who had both low air pollution—defined as less than or equal to the 75th percentile—and low NDI—defined as less than or equal to the 75th percentile. ORs or PRs for each of the other three exposure groups (high air pollution and low NDI, low air pollution and high NDI, and high air pollution and high NDI) are all presented in relation to the same low-low reference group. A *p*-value < 0.05 for the interaction term between air pollution and NDI (noted with an asterisk) was observed with NO_2_ for diabetes, stroke, and obesity and with PM_2.5_ for stroke.

The following model was used to calculate the ORs and PRs in Figure 1 and Figure 2, where the three exposure classification groups, “high-low”, “low-high”, and “high-high”, are coded using three indicator variables (adjustment for additional covariates not shown):

(2) $$logit\left\{P\left(Outcome=1|\begin{array}{c} high\;air\;pollution,\;low\;ACEs\;or\;NDI=a;\;\\ low\;air\;pollution,\;high\;ACEs\;or\;NDI=b;\;\\ high\;air\;pollution,\;high\;ACEs\;or\;NDI=c \end{array}\right)\right\}\;=\;\beta_{0}+\beta_{1}a+\beta_{2}b+\beta_{3}c$$

ORs or PRs were obtained by exponentiation of these coefficients from the logistic or log binomial and Poisson models, respectively. Each OR or PR had the same reference group: persons with low levels of both air pollution and the psychosocial stressor of interest, i.e., the “low-low” group. These ORs can be used to calculate the ratio of ORs to assess multiplicative interaction (see Supplementary Materials, Table S4). 

Additive interaction between air pollution and each of the stressors was also investigated. The relative excess risk due to interaction (RERI) was calculated as follows:

(3) $$RERI_{OR}=OR_{11}-OR_{10}-OR_{01}+1$$

As described by VanderWeele & Knol (2014), positive additive interaction (RERI >0) indicates that if an intervention were to reduce the first exposure, the public health impact would be larger in the group with a high level of the second exposure than in the group with a low level of the second exposure [37]. For example, an RERI greater than zero for the interaction between air pollution and ACEs would suggest that an intervention to reduce air pollution exposure would have a larger impact among those with high ACEs compared to those with low ACEs. Beyond the sign of the RERI (positive for positive interaction and negative for negative interaction), the numeric value of the RERI does not have an interpretation.

## 3. Results

The BRFSS survey was administered to 20,799, 20,551, and 14,769 respondents, in 2009, 2010, and 2011, respectively. To reduce participant burden and increase availability of data on other health issues, 36%, 67%, and 100% of those respondents, in 2009, 2010, and 2011, respectively, were asked about ACEs (*n* = 36,057). Respondents were excluded if they did not answer any questions about ACEs (*n* = 3397); if they did not have an air pollution exposure estimate due to a missing, incomplete, or non-residential zip code (*n* = 1088); or if ACS data were not available for that zip code to calculate NDI (*n* = 325). In total, 32,151 participants in 502 unique zip codes were included in this study. Participants with and without ACEs data were similar (data not shown).

Characteristics of participants in this study are shown in Table 1 for the entire sample, and by level of PM_2.5_, ACEs, and NDI. A descriptive table of participant characteristics by level of PM_10_ and NO_2_ is available in the Supplement (Table S1). Subjects who reported two or more ACEs tended to be younger and were more likely to be female; American Indian or Alaska Native, or multiracial; employed; a current or former smoker; and to report binge drinking, than those reporting zero or one ACEs. Subjects living in zip codes with a high NDI were less likely to be white, have some college education, or have an annual income of more than $35,000 per year, and more likely to be a current smoker, than subjects living in zip codes with a low NDI. 

Among all respondents, 11% reported diabetes, 4% reported stroke, 5% reported MI, 5% reported CHD, 39% reported hypertension, and 28% were classified as obese based on reported height and weight (Table 1). Diabetes, stroke, and obesity were more common among those with more ACEs and all six outcomes were more common among those with higher NDI. Diabetes and obesity were also more common among those exposed to high levels of some pollutants. Final results for CHD were similar to those for MI and results for hypertension were similar to obesity, and thus we present results for diabetes, stroke, MI, and obesity only.

ACEs scores ranged from 0 to 8, with a mean in this sample of 1.55 (standard deviation = 1.82). About 40% of the sample reported zero ACEs, 22% reported one ACE, 13% reported two ACEs and 25% reported three or more ACEs. The most commonly reported ACE was being a victim of verbal/emotional abuse (36.1% of subjects). The second most commonly reported ACE was living with someone with a substance abuse problem—a problem drinker or alcoholic, a user of illegal street drugs, or an abuser of prescription medications (29.1% of subjects). While the Spearman Rank correlation between ACEs and NDI was low (ρ = 0.04), a consistent trend of increasing neighborhood deprivation was observed across increasing ACEs score with a statistically significant difference across ACEs groups. Among those with 0 ACEs, 24% lived in zip codes with high NDI, whereas among those with 8 ACEs, 32% lived in zip codes with high NDI.

Air pollution levels were compared between those with low versus high ACEs and between those with low versus high NDI using *t*-tests (Table 2). Air pollution concentrations on average tended to be similar for those with 2 or more ACEs compared to those with 0 or 1 ACEs. In contrast, air pollution was statistically significantly different in high versus low NDI areas, with higher PM_10_ and NO_2_ in the group with high NDI. Pollution exposure was dichotomized at the 75th percentile, which was 7.6 µg/m^3^ for PM_2.5_, 15.5 µg/m^3^ for PM_10_, and 9.8 ppb for NO_2_. Both PM_2.5_ and NO_2_ tended to be higher in urban areas, whereas PM_10_ tended to be higher in the central and southeast rural portions of the state (Figure S1).

The main effect of ACEs on CVD outcomes and CVD risk factors was strong, even after adjustment for NDI and other covariates (e.g., ORs or PRs ranged from 1.3 for obesity (95% CI: 1.2, 1.3) to 1.6 for stroke (95% CI: 1.4, 1.8), Table S2). The main effect of NDI was also generally strong, even after adjustment for other covariates (e.g., ORs or PRs ranged from 1.2 for obesity (95% CI: 1.1, 1.3) to 1.3 for diabetes (95% CI: 1.2, 1.4), Table S2). Mostly null associations were observed for the main effect of high air pollution in fully adjusted models (data not shown).

### 3.1. Air Pollution and ACEs

Interaction analyses between air pollution and ACEs are presented in Figure 1. For each exposure and outcome combination, the figure shows the effect estimate for three groups of subjects in comparison to the same low air pollution-low ACEs reference group: those with high air pollution and low ACEs compared to the low air pollution-low ACEs reference group, those with low air pollution and high ACEs compared to the low air pollution-low ACEs reference group, and those with high air pollution and high ACEs compared to the low air pollution-low ACEs reference group. Each of the estimates presented are from the same model, adjusting for the same set of covariates. A detailed description and an example of how these ORs and PRs can be used to calculate estimates for the effect of air pollution exposure within strata of ACEs exposures can be found in the Supplementary Materials. 

Little multiplicative interaction was observed between air pollution and ACEs (Figure 1, Table S5). The interaction was statistically significant for the joint effect of PM_10_ and ACEs on diabetes (OR comparing high-high group to low-low reference group is 1.58 (95% CI: 1.40, 1.79); *p* < 0.05 for the multiplicative interaction term, Table S5). Those exposed to both high levels of PM_10_ and 2 or more ACEs had an elevated odds of diabetes compared to those with low levels of both exposures. The rest of the analyses shown in Figure 1 did not show a significant interaction between any of the three pollutants and ACEs. Similar results were observed when varying cutpoints were used to dichotomize ACEs (see Supplementary Materials, Table S3). Minimally adjusted and exploratory models also showed similar results, as did sensitivity analyses using the census-derived variable definition for urban versus rural (data not shown).

In addition to multiplicative interaction for PM_10_ and ACEs on diabetes, we also observed statistically significant positive additive interaction for this association. Table 3 presents the RERI for air pollution and ACEs in the fully adjusted model. An RERI of 0.26 (95% CI: 0.05, 0.48) indicates a positive additive interaction between PM_10_ and ACEs on diabetes, suggesting that a reduction in PM_10_ would have a greater impact on diabetes in the group with high ACEs scores than in the group with low ACEs scores.

### 3.2. Air Pollution and NDI

Multiplicative interaction was observed in some analyses of air pollution and NDI (Figure 2, Table S6). The odds of diabetes, stroke, and obesity are higher in high deprivation compared to low deprivation areas, with larger magnitudes of effect observed in areas that also have higher levels of ambient air pollution. For example, the odds of diabetes for those with high NO_2_ and high NDI was 1.40 (95% CI: 1.22, 1.60) times that compared to those with low NO_2_ and low NDI (*p* for interaction <0.05). The multiplicative interaction between air pollution and NDI was also statistically significant (*p* < 0.05) for the joint effect of NO_2_ and NDI on stroke and obesity, and for the joint effect of PM_2.5_ and NDI on stroke. Minimally adjusted and exploratory models showed similar results (data not shown).

A sensitivity analysis restricted to zip codes (*n* = 226) in more densely populated counties in the state (King, Pierce, Snohomish, and Spokane counties) yielded overall similar results to the analysis including all zip codes. In addition to significant interactions observed in the primary analysis, statistically significant multiplicative interaction was also observed between PM_10_ and NDI for the diabetes outcome when the analysis was restricted to these four counties (data available upon request). A sensitivity analysis using a census-derived definition of the urban versus rural covariate also yielded similar results to what is presented (data not shown). A sensitivity analysis was conducted using a multilevel model with a random intercept for zip code, and observed results were similar to the primary analysis.

Table 4 presents the additive interaction between air pollutants and NDI. For the stroke outcome, we observed positive additive interaction for both PM_2.5_ and NO_2_ with NDI (RERI of 0.39 (95% CI: 0.02, 0.77) and 0.37 (95% CI: 0.01, 0.74), respectively). For the diabetes outcome, we observed both multiplicative and positive additive interaction of NO_2_ and NDI.

## 4. Discussion

This study, which examines the interaction between psychosocial and environmental exposures, allows us to improve our understanding of how exposures across domains and across the life course produce negative health outcomes. This work adds to the growing body of literature on the influence of both early and later life stressors on physical disease outcomes [38,39,40]. Early life adversity may lead to a number of additional adversities later on in life, including limited financial resources and residing in a disadvantaged neighborhood. The interaction of psychosocial stressors with an ubiquitous environmental hazard investigated here further develops our understanding of the impact of multiple adversities on health.

Limited interaction, on either the additive or multiplicative scale, was seen between air pollution and ACEs. This result may be due to several limitations in exposure measurement of either air pollution or ACEs. First, ACEs and air pollution were assessed on differing spatial scales, at the individual and zip code levels, respectively. Generally weak air pollution effects were observed in this study, and this may in part be due to the use of larger geographic scale zip codes, at which to assess exposure rather than a more granular scale such as residential address used in many studies of air pollution and cardiovascular health [41]. This may be especially problematic in analyses of NO_2_, a marker of traffic-related air pollution that varies on a smaller spatial scale than other pollutants. Secondly, particulate matter is made up of a variety of compounds and this study was not able to examine the various components of PM_2.5_ separately. Third, differences across the life course in the development of coping mechanisms or in the experience of increasing adversity as a result of ACEs, may have limited the interaction with air pollution observed in this study. We were unable to account for the age at which the ACEs occurred or for differences in the time between occurrence of the ACEs and the air pollution exposure. This may result in a null effect if some subjects have stronger effects of more recent ACEs while other subjects have weaker effects as they develop coping mechanisms over time. Alternatively, ACE effects may become more fully exerted throughout adulthood through mediated pathways such as low income, which is often shared at a neighborhood level, and other adverse conditions in adulthood [42]. This may result in potentially stronger effects observed later in life with increasing cumulative adversity.

Furthermore, there may be varying effects of ACEs across the population, as some people will exhibit stronger health effects than others. Extreme stressors in early life, such as childhood poverty, may contribute to increased sensitization (i.e., lower levels of exposures are required for active stress responding) to the health effects of additional stressors later in life through dysregulation of biologic responses [43,44]. Different types of ACEs may also yield different effects on health or effects at different points across the life course [17,38]. The results presented here are consistent with the hypothesis that for some subjects there is increased vulnerability with more ACEs while for others there appears to be resilience and more limited effects on poor health. Alternatively, there simply may be no true interaction between air pollution and ACEs at the low level of air pollution seen in this study.

There was some evidence of multiplicative and positive additive interaction between air pollution and NDI on CVD-related outcomes. These interactions may be driven by the stronger associations between NDI and air pollution. Environmental justice research has repeatedly shown that disadvantaged neighborhoods tend to have higher levels of air pollution across the US [45]. Unlike ACEs, NDI and air pollution exposures were measured at the same spatial scale and may be influenced by similar upstream factors, including residential segregation [46]. Furthermore, adversity in childhood likely leads to increased risk of adversities in adulthood; that is, disadvantaged children such as those who experience ACEs are more likely to grow up as disadvantaged adults, living in disadvantaged neighborhoods with higher air pollution. Thus, we observe a stronger effect of the more proximal exposure, NDI, relative to ACEs when interacting with an environmental stressor [40,47].

The consistent association with diabetes may also reflect influential built environment characteristics captured in both the NDI and air pollution metrics, as more extensive built environments are likely more urban and thus more likely to have higher concentrations of pollution, particularly traffic-related air pollution such as NO_2_. In several models, we also observed a protective effect of air pollution exposure, particularly NO_2_, among subjects with low NDI; this is not unexpected based on prior work [9]. We hypothesize that this is due to the impact of additional individual SES factors and other buffering effects found among those living in low NDI, and thus generally high SES, neighborhoods [17].

The prior epidemiologic evidence of interactions between stressors and air pollution on CVD outcomes is largely null [48,49]. Hicken et al. did not find synergistic effects of acute exposure to PM_2.5_ and various psychosocial stressors on blood pressure in the Multi-Ethnic Study of Atherosclerosis [50]. In the same study population, the association between PM_2.5_ and left ventricular mass index was stronger in blacks compared to whites, but there was no evidence of interaction between PM_2.5_ and other markers of social disadvantage such as, individual level SES, racial residential segregation and a composite variable representing psychological adversity [7]. In the Detroit Healthy Environments Partnership, a larger magnitude of effect of acute PM_2.5_ on systolic blood pressure was found among those reporting high levels of stress (a composite index of several stressors), but this interaction was only statistically significant in one of the three study areas [51].

Early life stress induces changes in multiple physiologic systems, including nervous, endocrine, and immune systems [44]. These changes can contribute to the proliferation of additional stress exposures and to cumulative effects of chronic stress leading to increased risk of disease in adulthood [52]. The epidemiologic literature on mental health outcomes is generally consistent with these conceptual models; the effect of later-life stressors on mental health outcomes such as depression is larger among those who had also experienced early-life stressors [53,54]. This framework is also posited for later environmental insults to the body resulting not only in poor mental health, but in poor physical health as well [12,13,15,55]. Specifically, we hypothesized that synergistic deleterious health effects on such shared pathways may occur in the presence of both higher air pollution exposure and higher stress. 

The results presented here should be interpreted in the context of the limitations of this study. First, air pollution exposures were assessed on a large spatial scale, which likely contributes to measurement error. Additionally, using exposures at this scale reduced the variability of air pollution exposure, but zip code was the smallest geography available for analysis of BRFSS participants. However, measurement error resulting from exposure assessment on this spatial scale is likely less problematic for pollutants such as particulate matter with large-scale regional distribution patterns. Similar to prior literature, annual average exposures were used as proxies for longer time periods [7,56]. Second, the health outcomes were self-reported and thus there may be self-report bias, particularly in reporting weight. However, reports of some outcomes such as diabetes may be less subject to recall bias because the outcome is diagnosis of a specific disease such as diabetes, rather than recall of an everyday event such as average number of minutes of physical activity per day. There may also be bias in reporting ACEs, such as older adults reporting fewer ACEs [38]. Lastly, this study relied on cross-sectional data. While a large suite of covariates was included in the model, it is possible that there was unmeasured confounding, specifically at the contextual level.

However, there were also several strengths to this study. The large sample size with data on psychosocial stressors in childhood is a major strength of this study. Additionally, ACEs are chronic stressors that are almost always perceived as negative, and thus there is little to no uncertainty about stress perception with this exposure. Few studies have reported additive interactions as well as interactions on the multiplicative scale. As seen in this analysis there may be an additive effect, even in the absence of a multiplicative one, which is relevant for informing public health policy. Furthermore, we were able to examine both an individual-level stressor and a neighborhood-level stressor in the same population, which emphasizes the importance of the broader context in which disease develops. Lastly, although not a longitudinal study, we were able to evaluate stressors over the life course to better understand the role of both early childhood stress and later life stressors on adult health outcomes.

## 5. Conclusions

The evidence is growing for impacts of both psychosocial stressors and air pollution on similar biologic pathways such as immune function, inflammatory response, and sympathetic nervous system [4,44]. However, some epidemiologic studies, including this one, have observed only modest evidence of interactions between air pollution and stress. Both social and environmental determinants of health are responsible for the creation and persistence of health disparities across place and time. Thus, our study provides some insight into the interaction between these two important determinants and the role of cumulative risk in the development of CVD.

## Figures and Tables

**Table 1:** WA State 2009–2011 BRFSS participant characteristics overall, and by PM_2.5_ exposure, ACEs score, or NDI ^a^.

Participant Characteristic	Overall (*n* = 32,151)		Low PM_2.5_^b^ (*n* = 24,115)		High PM_2.5_^b^ (*n* = 8036)		≤1 ACE (*n* = 19,941)		2+ ACEs (*n* = 12,210)		Low NDI ^c^ (*n* = 24,193)		High NDI ^c^ (*n* = 7958)	
Age ^d^	57	(16)	57	(16)	56	(16)	59	(16)	53	(15)	57	(16)	56	(17)
Female	19,420	(60)	14,536	(60)	4884	(61)	11,684	(59)	7736	(63)	14,593	(60)	4827	(61)
Race/ethnicity														
White	27,878	(87)	20,989	(87)	6889	(86)	17,495	(88)	10,383	(85)	21,470	(89)	6408	(81)
Hispanic	1534	(5)	1078	(4)	456	(6)	907	(5)	627	(5)	743	(3)	791	(10)
Black	383	(1)	260	(1)	123	(2)	214	(1)	169	(1)	224	(0.9)	159	(2)
Asian	691	(2)	505	(2)	186	(2)	574	(3)	117	(1)	582	(2)	109	(1)
American Indian, Alaska Native	314	(1)	250	(1)	64	(0.8)	118	(0.6)	196	(2)	201	(0.8)	113	(1)
Native Hawaiian, Pacific Islander	90	(0.3)	60	(0.3)	30	(0.4)	53	(0.3)	37	(0.3)	60	(0.3)	30	(0.4)
Multiracial	769	(2)	588	(2)	181	(2)	292	(1)	477	(4)	552	(2)	217	(3)
Other	191	(0.6)	155	(0.6)	36	(0.5)	100	(0.5)	91	(0.8)	135	(0.6)	56	(0.7)
Education														
Less than HS	1719	(5)	1287	(5)	432	(5)	955	(5)	764	(6)	912	(4)	807	(10)
HS	7270	(23)	5495	(23)	1775	(22)	4548	(23)	2722	(22)	4928	(20)	2342	(29)
Some college	23,123	(72)	17,304	(72)	5819	(73)	14,409	(72)	8714	(71)	18,327	(76)	4796	(60)
Annual income														
<$35,000	9648	(34)	7189	(34)	2459	(35)	5752	(33)	3896	(36)	6599	(31)	3049	(44)
$35,000–$49,999	4756	(17)	3567	(17)	1189	(17)	2964	(17)	1792	(17)	3511	(16)	1245	(18)
Employed	15,793	(49)	11,739	(49)	4054	(51)	9350	(47)	6443	(53)	12,068	(50)	3725	(47)
Smoking														
Current	4282	(13)	2181	(13)	1101	(14)	1942	(9)	2340	(19)	2930	(12)	1352	(17)
Former	10,300	(32)	7821	(33)	2479	(31)	6075	(31)	4225	(35)	7788	(32)	2512	(32)
Binge Drinking	3809	(12)	2908	(12)	901	(11)	1989	(10)	1820	(15)	2907	(12)	902	(11)
BMI (kg/m^2^) ^d^	28	(6)	28	(6)	28	(6)	27	(6)	28	(6)	27	(6)	28	(6)
CVD and CVD risk factors														
Diabetes	3685	(11)	2705	(11)	980	(12)	2238	(11)	1447	(12)	2590	(11)	1095	(14)
Stroke	1178	(4)	865	(4)	313	(4)	688	(3)	490	(4)	817	(3)	361	(5)
MI	1573	(5)	1201	(5)	372	(5)	986	(5)	587	(5)	1089	(5)	484	(6)
CHD	1699	(5)	1309	(5)	390	(5)	1057	(5)	642	(5)	1225	(5)	474	(6)
Obesity	8460	(28)	6001	(40)	1745	(37)	4711	(25)	3749	(32)	6028	(26)	2432	(32)
Hypertension ^e^	7746	(39)	6298	(27)	2162	(28)	4931	(40)	2815	(38)	5765	(39)	1981	(41)

PM_2.5_: fine particulate matter, ACEs: adverse childhood experiences, NDI: neighborhood deprivation index. ^a^ Values expressed as number (%), unless otherwise specified. ^b^ Low PM_2.5_ exposure defined as a concentration less than or equal to 7.55 µg/m^3^ (the 75th percentile) and high exposure was defined as greater than the 75th percentile. ^c^ Low NDI is defined as less than or equal to the 75th percentile of the index within this sample, which was 1.64. High NDI is defined as the 25th percentile with the highest deprivation. ^d^ Values for these variables expressed as mean (standard deviation). ^e^ Hypertension was only reported in 2009 and 2011 (*n* = 19,679).

**Table 2:** Mean (standard deviation) of PM_2.5_, PM_10_, and NO_2_ exposures, by ACEs score and NDI.

Overall	PM_2.5_ (µg/m^3^)		PM_10_ (µg/m^3^)		NO_2_ (ppb)	
	6.54	(1.41)	13.95	(2.97)	7.50	(2.86)
**ACEs ^a^**						
Low	6.56	(1.40)	13.43	(2.48)	7.48	(2.82)
High	6.53	(1.44)	13.42	(2.47)	7.52	(2.91)
*p*	0.20		0.71		0.25	
**NDI ^b^**						
Low	6.57	(1.35)	13.33	(2.36)	7.48	(2.71)
High	6.45	(1.58)	13.70	(2.78)	7.56	(3.26)
*p*	<0.01		<0.01		0.03	

PM_2.5_: fine particulate matter, PM_10_: particulate matter less than 10 µm in diameter, NO_2_: nitrogen dioxide, ACEs: adverse childhood experiences, NDI: neighborhood deprivation index. ^a^ Low ACEs score was defined as 0 or 1 ACEs. High ACEs score was defined as 2 or more ACEs. ^b^ Low NDI is defined as less than or equal to the 75th percentile of the index within this sample, which was 1.64. High NDI is defined as the 25th percentile with the highest deprivation.

**Table 3:** Relative excess risk due to interaction (RERI) between air pollution and ACEs on CVD-related outcomes ^a,b^.

Outcome	PM_2.5_ and ACEs		PM_10_ and ACEs		NO_2_ and ACEs	
Diabetes	0.02	(−0.22, 0.25)	0.26	(0.05, 0.48) *	0.09	(−0.13, 0.30)
Stroke	0.02	(−0.40, 0.44)	−0.06	(−0.44, 0.31)	0.14	(−0.27, 0.55)
MI	−0.10	(−0.44, 0.24)	−0.02	(−0.33, 0.30)	−0.10	(−0.44, 0.25)
Obesity	−0.03	(−0.18, 0.13)	−0.08	(−0.23, 0.07)	−0.01	(−0.16, 0.14)

^a^ PM_2.5_: fine particulate matter, PM_10_: particulate matter less than 10 µm in diameter, NO_2_: nitrogen dioxide, ACEs: adverse childhood experiences, MI: myocardial infarction. ^b^ RERI_OR_ (relative excess risk due to interaction) is calculated as (OR_11_) − (OR_10_) − (OR_01_) + 1 where subscript 11 designates high air pollution and high ACEs, subscript 10 designates the group with high air pollution and low ACEs, and subscript 01 designates the group with low air pollution and high ACEs. Models are adjusted for age, race, sex, education, employment, income, urban versus rural zip code and neighborhood deprivation index (NDI). Estimates with a *p*-value < 0.05 are indicated with an asterisk.

**Table 4:** Relative excess risk due to interaction (RERI) between air pollution and NDI on CVD-related outcomes ^a,b^.

Outcome	PM_2.5_ and NDI		PM_10_ and NDI		NO_2_ and NDI	
Diabetes	0.22	(−0.02, 0.46)	0.20	(−0.03, 0.42)	0.47	(0.26, 0.69) *
Stroke	0.39	(0.02, 0.77) *	−0.06	(−0.41, 0.28)	0.37	(0.01, 0.74) *
MI	0.22	(−0.12, 0.55)	0.02	(−0.29, 0.33)	0.24	(−0.09, 0.58)
Obesity	0.13	(−0.04, 0.30)	0.08	(−0.08, 0.24)	0.15	(−0.01, 0.31)

^a^ PM_2.5_: fine particulate matter, PM_10_: particulate matter less than 10 µm in diameter, NO_2_: nitrogen dioxide, NDI: neighborhood deprivation index, MI: myocardial infarction. ^b^ RERI_OR_ (relative excess risk due to interaction) is calculated as (OR_11_) − (OR_10_) − (OR_01_) + 1 where subscript 11 designates high air pollution and high NDI, subscript 10 designates the group with high air pollution and low NDI, and subscript 01 designates the group with low air pollution and high NDI. Models are adjusted for age, race, sex, education, employment, income and urban versus rural zip code. Estimates with a *p*-value < 0.05 are indicated with an asterisk.

## Supplementary Materials

The following are available online at http://www.mdpi.com/1660-4601/15/3/472/s1, Figure S1: Map of air pollution exposures for 2009–2011 BRFSS participants, averaged at the zip code level in Washington State, Table S1: Mean (standard deviation) or number (percent) of WA State 2009–2011 BRFSS participant characteristics by PM_10_ or NO_2_ exposure, Table S2: Odds ratios or prevalence ratios (95% confidence intervals) for main effects models of adverse childhood experiences (ACEs) and neighborhood deprivation index (NDI) on cardiovascular-related outcomes, Table S3: Odds ratios or prevalence ratios (95% confidence intervals) for the relationship between air pollutants (PM_2.5_, PM_10_, and NO_2_), adverse childhood experiences (ACEs), and cardiovascular-related outcomes, using varying dichotomizations of ACEs, Table S4: Example of how odds ratios for air pollution exposure are calculated within each strata of the stressor, given the odds ratios with regards to a single reference group, Table S5: Estimates of air pollution effects on cardiovascular disease (CVD) measures and risk factors, within strata of adverse childhood experiences (ACEs) and estimates of multiplicative interaction (ratio of odds ratios or ratio of prevalence ratios), Table S6: Estimates of air pollution effects on cardiovascular disease (CVD) measures and risk factors, within strata of Neighborhood Deprivation Index (NDI) and estimates of multiplicative interaction (ratio of odds ratios or ratio of prevalence ratios).
