# Supplementary material for: Individual and Neighborhood Stressors, Air Pollution and Cardiovascular Disease
Source: Int J Environ Res Public Health. 2018 Mar 8;15(3):472. doi: 10.3390/ijerph15030472 (PMC5877017; doi:10.3390/ijerph15030472)
Supplement: Supplementary file 1 [file ijerph-15-00472-s001.docx]

Supplementary Material

**Table S1.** Mean (standard deviation) or number (percent) of WA State 2009–2011 BRFSS participant characteristics by PM_10_ or NO_2_ exposure.

| **Participant Characteristic** | **Low PM_10_ ^a^** | | **High PM_10_ ^a^** | | **Low NO_2_ ^a^** | | **High NO_2_ ^a^** | |
| --- | --- | --- | --- | --- | --- | --- | --- | --- |
| Age, mean (SD) | 57 | (16) | 56 | (16) | 57 | (16) | 56 | (16) |
| Female, *n* (%) | 12,916 | (60) | 4,373 | (61) | 14,614 | (61) | 4,806 | (60) |
| Race/ethnicity, *n* (%) |  | | | | | | | |
| White | 18,742 | (87) | 6,253 | (87) | 21,187 | (88) | 6,691 | (83) |
| Hispanic | 785 | (4) | 361 | (5) | 1,121 | (5) | 413 | (5) |
| Black | 275 | (1) | 94 | (1) | 174 | (0.7) | 209 | (3) |
| Asian | 544 | (3) | 128 | (2) | 369 | (2) | 322 | (4) |
| American Indian or Alaska Native | 223 | (1) | 69 | (1) | 255 | (1) | 59 | (0.7) |
| Native Hawaiian or Pacific Islander | 61 | (0.3) | 22 | (0.3) | 61 | (0.3) | 29 | (0.4) |
| Multiracial | 540 | (3) | 163 | (2) | 578 | (2) | 191 | (2) |
| Other | 137 | (0.6) | 32 | (0.5) | 144 | (0.6) | 47 | (0.6) |
| Education, *n* (%) |  | | | | | | | |
| Less than HS | 945 | (4) | 414 | (6) | 1,352 | (6) | 367 | (5) |
| HS | 4,716 | (22) | 1,641 | (23) | 5,688 | (24) | 1,582 | (20) |
| Some college | 15,833 | (74) | 5,112 | (71) | 17,047 | (71) | 6,076 | (76) |
| Annual income, *n* (%) |  | | | | | | | |
| <$35,000 | 6,194 | (33) | 2,273 | (36) | 7,351 | (35) | 2,297 | (33) |
| $35,000-$49,999 | 3,123 | (17) | 1,105 | (18) | 3,648 | (17) | 1,108 | (16) |
| Employed, *n* (%) | 10,464 | (49) | 3,642 | (51) | 11,616 | (48) | 4,177 | (52) |
| Smoking, *n* (%) |  | | | | | | | |
| Current | 2,817 | (13) | 1,037 | (15) | 3,227 | (13) | 1,055 | (13) |
| Former | 7,058 | (33) | 2,249 | (31) | 7,765 | (32) | 2,535 | (32) |
| Binge Drinking, *n* (%) | 2,592 | (12) | 829 | (12) | 2,840 | (12) | 969 | (12) |
| BMI (kg/m^2^), mean (SD) | 28 | (6) | 28 | (6) | 28 | (6) | 27 | (6) |
| CVD and CVD risk factors |  | | | | | | | |
| Diabetes | 2,434 | (11) | 1,251 | (12) | 2,796 | (12) | 889 | (11) |
| Stroke | 800 | (4) | 378 | (4) | 877 | (4) | 301 | (4) |
| MI | 1,059 | (5) | 514 | (5) | 1,203 | (5) | 370 | (5) |
| CHD | 1,199 | (6) | 500 | (5) | 1,301 | (5) | 398 | (5) |
| Hypertension ^b^ | 5,244 | (40) | 2,502 | (38) | 5,707 | (40) | 2,039 | (37) |
| Obesity | 5,652 | (27) | 2,808 | (29) | 6,432 | (28) | 2,028 | (26) |

^a^ Low air pollution defined as a concentration less than or equal to the 75^th^ percentile and high air pollution was defined as greater than the 75^th^ percentile. The 75^th^ percentile was 14.95 µg/m^3^ for PM_10_, and 9.79 ppb for NO_2_. ^b^ Hypertension was only reported in 2009 and 2011 (*n* = 19,679).

**Table S2.** Odds ratios or prevalence ratios (95% confidence intervals) for main effects models of adverse childhood experiences (ACEs) and neighborhood deprivation index (NDI) on cardiovascular-related outcomes ^a^.

| **Outcome** | **ACEs ^b^** | | | **NDI** |
| --- | --- | --- | --- | --- |
|  | **0 versus 1+** | **0-1 versus 2+** | **0-2 versus 3+** | **≥75^th^%. versus <75^th^ %.** |
| Diabetes | 1.32 (1.21, 1.43) | 1.36 (1.26, 1.48) | 1.39 (1.27, 1.52) | 1.28 (1.17, 1.39) |
| Stroke | 1.46 (1.27, 1.68) | 1.56 (1.36, 1.79) | 1.84 (1.58, 2.14) | 1.24 (1.07, 1.43) |
| MI | 1.29 (1.15, 1.46) | 1.35 (1.20, 1.53) | 1.61 (1.41, 1.85) | 1.27 (1.12, 1.44) |
| Obesity ^c^ | 1.25 (1.19, 1.31) | 1.25 (1.19, 1.31) | 1.23 (1.17, 1.29) | 1.19 (1.14, 1.25) |

^a^ Models adjusted for age, race, sex, urbanicity, education, income, and employment. ^b^ Models of ACEs additionally adjusted for NDI. ^c^ Prevalence ratios.

**Table S3.** Odds ratios or prevalence ratios (95% confidence intervals) for the relationship between air pollutants (PM_2.5_, PM_10_, and NO_2_), adverse childhood experiences (ACEs), and cardiovascular-related outcomes, using varying dichotomizations of ACEs ^a^.

| **Outcome** | **ACEs** | **PM_2.5_** | | **PM_10_** | | **NO_2_** | |
| --- | --- | --- | --- | --- | --- | --- | --- |
|  |  | **<75^th^ %.** | **≥75^th^ %.** | **<75^th^ %.** | **≥75^th^ %.** | **<75^th^ %.** | **≥75^th^ %.** |
| Diabetes | 0 | *ref* | 1.20 (1.04, 1.38) | *ref* | 0.99 (0.87, 1.13) | *ref* | 0.89 (0.76, 1.03) |
|  | 1-8 ^b^ | 1.36 (1.24, 1.50) | 1.43 (1.26, 1.62) | 1.25 (1.13, 1.38) | 1.46 (1.30, 1.64) | 1.29 (1.18, 1.42) | 1.26 (1.11, 1.42) |
|  | 0-2 | *ref* | 1.12 (1.01, 1.24) | *ref* | 1.06 (0.97, 1.17) | *ref* | 0.91 (0.82, 1.01) |
|  | 3-8 ^c^ | 1.40 (1.26, 1.55) | 1.51 (1.29, 1.77) | 1.33 (1.19, 1.48) | 1.61 (1.40, 1.86) ^d^ | 1.34 (1.21, 1.49) | 1.39 (1.18, 1.62) |
| Stroke | 0 | *ref* | 0.98 (0.76, 1.26) | *ref* | 0.87 (0.69, 1.09) | *ref* | 1.04 (0.81, 1.33) |
|  | 1-8 | 1.40 (1.20, 1.64) | 1.53 (1.24, 1.87) | 1.37 (1.16, 1.62) | 1.38 (1.14, 1.69) | 1.43 (1.22, 1.68) | 1.51 (1.23, 1.86) |
|  | 0-2 | *ref* | 1.00 (0.84, 1.20) | *ref* | 0.94 (0.80, 1.12) | *ref* | 1.05 (0.88, 1.26) |
|  | 3-8 | 1.74 (1.46, 2.07) | 2.09 (1.63, 2.70) | 1.80 (1.50, 2.15) | 1.78 (1.39, 2.27) | 1.83 (1.54, 2.18) | 1.89 (1.46, 2.45) |
| MI | 0 | *ref* | 0.95 (0.76, 1.19) | *ref* | 0.88 (0.72, 1.07) | *ref* | 0.97 (0.78, 1.21) |
|  | 1-8 | 1.31 (1.15, 1.51) | 1.26 (1.04, 1.52) | 1.26 (1.09, 1.46) | 1.25 (1.05, 1.49) | 1.30 (1.14, 1.49) | 1.32 (1.09, 1.59) |
|  | 0-2 | *ref* | 0.98 (0.84, 1.15) | *ref* | 0.93 (0.80, 1.07) | *ref* | 0.99 (0.84, 1.16) |
|  | 3-8 | 1.67 (1.43, 1.95) | 1.50 (1.16, 1.93) | 1.59 (1.35, 1.87) | 1.60 (1.28, 2.00) | 1.62 (1.39, 1.90) | 1.64 (1.28, 2.10) |
| Obesity ^e^ | 0 | *ref* | 1.00 (0.91, 1.10) | *ref* | 1.05 (0.97, 1.14) | *ref* | 0.94 (0.86, 1.04) |
|  | 1-8 | 1.24 (1.17, 1.31) | 1.25 (1.17, 1.35) | 1.27 (1.20, 1.35) | 1.26 (1.17, 1.35) | 1.24 (1.17, 1.31) | 1.20 (1.12, 1.29) |
|  | 0-2 | *ref* | 1.01 (0.95, 1.08) | *ref* | 1.00 (0.95, 1.06) | *ref* | 0.96 (0.90, 1.02) |
|  | 3-8 | 1.22 (1.15, 1.30) | 1.24 (1.13, 1.36) | 1.21 (1.14, 1.29) | 1.25 (1.15, 1.35) | 1.22 (1.15, 1.29) | 1.19 (1.09, 1.31) |

^a^ Models were adjusted for age, race sex, urbanicity, education, income, employment, and neighborhood deprivation index. ^b^ High ACEs is defined as having 1 or more ACEs (*n* = 19,381). ^c^ High ACEs is defined as having 3 or more ACEs (*n* = 7,890). ^d^ Interaction p-value = 0.0484; ^e^ Prevalence ratios.

Odds Ratios within Strata and Multiplicative Interaction

Estimates of the effect of air pollution within ACEs or NDI strata, or estimates of the effect of ACEs or NDI within air pollution strata, can be calculated from the information provided in Figures 1 and 2. The formulas for calculating these odds ratios (ORs) are shown in Table S4. The inner 2 x 2 table shows ORs for the “high-low,” “low-high,” and “high-high” groups, each compared to the same “low-low” reference group as in Figures 1 and 2. For example, in Figure 1 the ORs for the high-low, low-high, and high-high groups in the analysis of the interaction between PM_2.5_ and ACEs on diabetes are 1.11, 1.37, and 1.49, respectively. The estimate of the effect of PM_2.5_ within the strata with low ACEs is 1.11, and within the strata with high ACEs, is 1.49/1.37 = 1.09. Effects of air pollution exposure within strata of ACEs and NDI are presented in Table S5.

The ORs in Figures 1 and 2 can also be used to calculate the multiplicative interaction term—the ratio of odds ratios—using the formula in the bottom right cell of Table S4, which is commonly presented in the literature. For example, the multiplicative interaction between PM_2.5_ and ACEs on diabetes is 1.49/(1.37*1.11) = 1.21. The p-values for the multiplicative interaction term are included for each analysis in Table S5.

**Table S4.** Example of how odds ratios for air pollution exposure are calculated within each strata of the stressor, given the odds ratios with regards to a single reference group ^a^.

| **Stressor** | **Low Air Pollution** | **High Air Pollution** | **OR for effect of air pollution within strata of the stressor** |
| --- | --- | --- | --- |
| Low stressor | OR_00_ = 1 *(ref)* | OR_10_ | OR_10_/1 |
| High stressor | OR_01_ | OR_11_ | OR_11_/OR_01_ |
|  |  |  | OR_11_/OR_10_OR_01_ ^b^ |

^a^ Odds ratios OR_11_, OR_10_, OR_01_ correspond to the odds ratios reported in Figures 1 and 2. ^b^ Measure of interaction on multiplicative scale: ratio of ORs.

**Table S5.** Estimates of air pollution effects on cardiovascular disease (CVD) measures and risk factors, within strata of adverse childhood experiences (ACEs) and estimates of multiplicative interaction (ratio of odds ratios or ratio of prevalence ratios).

| **Outcome** | **Strata of stressor** | **PM_2.5_** | **PM_10_** | **NO_2_** |
| --- | --- | --- | --- | --- |
| Diabetes | Low ACEs ^a^ | 1.11 (0.99, 1.25) | 1.03 (0.93, 1.15) | 0.91 (0.80, 1.02) |
|  | High ACEs ^a^ | 1.09 (0.95, 1.26) | 1.23 (1.08, 1.40) | 0.99 (0.86, 1.14) |
|  | *Ratio of ORs* | *0.98 (0.82, 1.17)* | *1.19 (1.01, 1.41)* | *1.10 (0.91, 1.32)* |
|  | *Interaction p-value* ^b^ | *0.82* | *0.04* | *0.33* |
| Stroke | Low ACEs | 1.06 (0.87, 1.28) | 0.97 (0.81, 1.16) | 1.01 (0.83, 1.23) |
|  | High ACEs | 1.05 (0.83, 1.33) | 0.94 (0.75, 1.17) | 1.10 (0.87, 1.39) |
|  | *Ratio of ORs* | *0.99 (0.74, 1.34)* | *0.97 (0.73, 1.29)* | *1.09 (0.81, 1.47)* |
|  | *Interaction p-value* | *0.96* | *0.83* | *0.57* |
| MI | Low ACEs | 0.99 (0.83, 1.17) | 0.95 (0.81, 1.11) | 1.02 (0.86, 1.22) |
|  | High ACEs | 0.92 (0.73, 1.15) | 0.95 (0.77, 1.17) | 0.95 (0.76, 1.19) |
|  | *Ratio of ORs* | *0.93 (0.70, 1.23)* | *1.00 (0.78, 1.30)* | *0.93 (0.70, 1.23)* |
|  | *Interaction p-value* | *0.61* | *0.98* | *0.60* |
| Obesity | Low ACEs | 1.02 (0.95, 1.10) | 1.03 (0.97, 1.10) | 0.96 (0.89, 1.03) |
|  | High ACEs | 0.99 (0.92, 1.08) | 0.98 (0.91, 1.05) | 0.97 (0.89, 1.05) |
|  | *Ratio of PRs* | *0.97 (0.88, 1.08)* | *0.95 (0.86, 1.04)* | *1.01 (0.91, 1.12)* |
|  | *Interaction p-value* | *0.61* | *0.27* | *0.89* |

^a^ Low ACEs defined as 0 or 1 ACEs. High ACEs defined as 2 or more ACEs. ^b^ P-value for multiplicative interaction.

**Table S6.** Estimates of air pollution effects on cardiovascular disease (CVD) measures and risk factors, within strata of Neighborhood Deprivation Index (NDI) and estimates of multiplicative interaction (ratio of odds ratios or ratio of prevalence ratios).

| **Outcome** | **Strata of stressor** | **PM_2.5_** | **PM_10_** | **NO_2_** |
| --- | --- | --- | --- | --- |
| Diabetes | Low NDI ^a^ | 1.08 (0.97, 1.20) | 1.10 (0.99, 1.21) | 0.78 (0.70, 0.88) |
|  | High NDI ^a^ | 1.24 (1.06, 1.45) | 1.24 (1.07, 1.44) | 1.23 (1.05, 1.43) |
|  | *Ratio of ORs* | *1.16 (0.96, 1.39)* | *1.13 (0.95, 1.35)* | *1.56 (1.29, 1.89)* |
|  | *Interaction p-value* ^b^ | *0.13* | *0.17* | *<0.01* |
| Stroke | Low NDI | 0.95 (0.79, 1.14) | 0.98 (0.83, 1.16) | 0.92 (0.76, 1.12) |
|  | High NDI | 1.30 (1.02, 1.67) | 0.93 (0.73, 1.19) | 1.26 (0.98, 1.62) |
|  | *Ratio of ORs* | *1.38 (1.01, 1.87)* | *0.95 (0.71, 1.28)* | *1.37 (1.00, 1.87)* |
|  | *Interaction p-value* | *0.04* | *0.75* | *0.05* |
| MI | Low NDI | 0.91 (0.77, 1.08) | 0.96 (0.83, 1.12) | 0.90 (0.75, 1.07) |
|  | High NDI | 1.11 (0.88, 1.40) | 0.98 (0.79, 1.23) | 1.12 (0.89, 1.41) |
|  | *Ratio of ORs* | *1.21 (0.91, 1.62)* | *1.02 (0.78, 1.34)* | *1.25 (0.94, 1.66)* |
|  | *Interaction p-value* | *0.18* | *0.87* | *0.13* |
| Obesity | Low NDI | 1.00 (0.95, 1.06) | 1.02 (0.97, 1.07) | 0.91 (0.86, 0.97) |
|  | High NDI | 1.07 (0.99, 1.15) | 1.05 (0.98, 1.13) | 1.02 (0.94, 1.10) |
|  | *Ratio of PRs* | *1.07 (0.97, 1.17)* | *1.04 (0.95, 1.13)* | *1.11 (1.01, 1.22)* |
|  | *Interaction p-value* | *0.16* | *0.44* | *0.03* |

^a^ Low NDI defined as less than or equal to the 75^th^ percentile. High NDI defined as greater than the 75^th^ percentile. ^b^ P-value for multiplicative interaction.

| **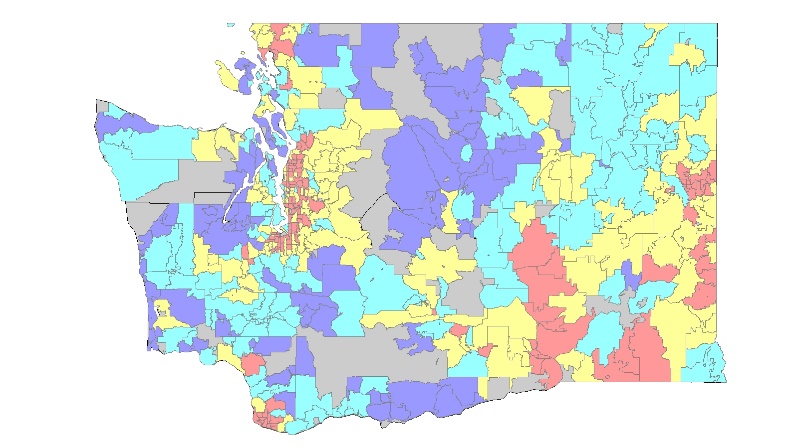** | **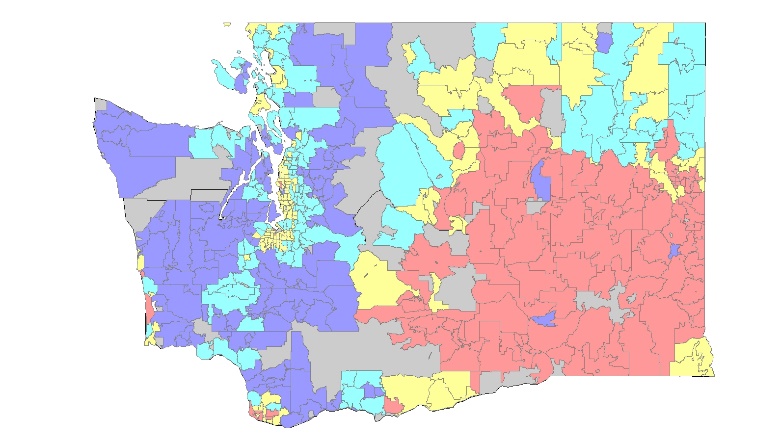** |
| --- | --- |
| (**a**) | (**b**) |
| **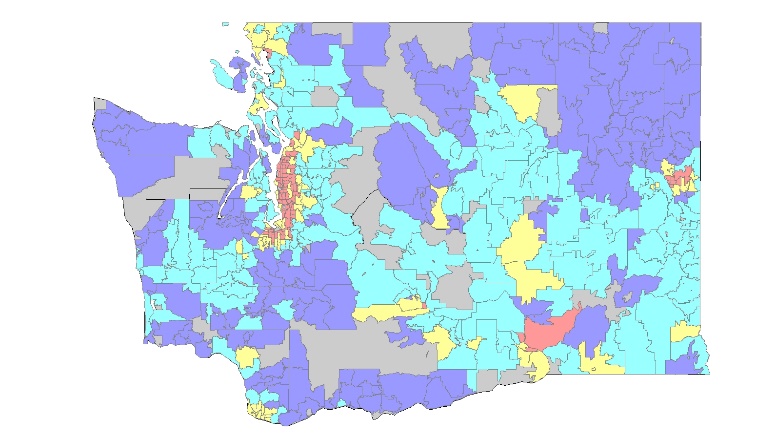** | |
| (**c**) | |

**Figure S1.** Map of air pollution exposures for 2009–2011 BRFSS participants, averaged at the zip code level in Washington State. Maps show spatial distribution across the state of (**a**) PM_2.5_, (**b**) PM_10_, and (**c**) NO_2_. Grey zip codes did not have participants included in this analysis.
